# The Role of Learning Support and Chat-Sessions in Guided Internet-Based Cognitive Behavioral Therapy for Adolescents With Anxiety: A Factorial Design Study

**DOI:** 10.3389/fpsyt.2020.00503

**Published:** 2020-06-10

**Authors:** Matilda Berg, Alexander Rozental, Josefine de Brun Mangs, Maja Näsman, Karin Strömberg, Linn Viberg, Erik Wallner, Hanna Åhman, Kristin Silfvernagel, Maria Zetterqvist, Naira Topooco, Andrea Capusan, Gerhard Andersson

**Affiliations:** ^1^ Department of Behavioural Sciences and Learning, Linköping University, Linköping, Sweden; ^2^ Department of Clinical Neuroscience, Karolinska Institutet, Stockholm, Sweden; ^3^ Institute of Child Health, University College London, London, United Kingdom; ^4^ Department of Clinical and Experimental Medicine (IKE), Center for Social and Affective Neuroscience, Linköping University, Linköping, Sweden; ^5^ Department of Child and Adolescent Psychiatry, Region Östergötland, Linkoöping, Sweden; ^6^ Center for m^2^Health, Palo Alto, CA, United States

**Keywords:** internet-based cognitive behavioral therapy, adolescents, anxiety, learning support, chat-sessions

## Abstract

**Background:**

Increased awareness of anxiety in adolescents emphasises the need for effective interventions. Internet-based cognitive behavioural therapy (ICBT) could be a resource-effective and evidence-based treatment option, but little is known about how to optimize ICBT or which factors boost outcomes. Recently, the role of knowledge in psychotherapy has received increased focus. Further, chat-sessions are of interest when trying to optimize ICBT for youths. This study aimed to evaluate the role of learning support and chat-sessions during ICBT for adolescent anxiety, using a factorial design.

**Method:**

A total of 120 adolescents were randomised to one of four treatment groups, in a 2x2 design with two factors: with or without learning support and/or chat-sessions.

**Results:**

Anxiety and depressive symptoms were reduced (Beck Anxiety Inventory- BAI; Cohen’s *d* =0.72; Beck Depression Inventory- BDI; *d* =0.97). There was a main effect of learning support on BAI (*d* =0.38), and learning support increased knowledge gain (*d* =0.42). There were no main effects or interactions related to the chat-sessions. Treatment effects were maintained at 6-months, but the added effect of learning support had by then vanished.

**Conclusion:**

ICBT can be an effective alternative when treating adolescents with anxiety. Learning support could be of importance to enhance short-term treatment effects, and should be investigated further.

## Introduction

Adolescence has been described as a time of vulnerability to anxiety (1), and in those affected, the risk of relapse and development of psychiatric problems later on in life is high (2–4). Youth anxiety is often associated with youth depression (5, 6). Epidemiological studies (7, 8) show that about 11% of all children and adolescents have some form of anxiety disorder. It is therefore important to provide effective and accessible treatments. Internet-based cognitive behavioral therapy (ICBT) is an evidence-based and often cost-effective form of treatment. Meta-analyses show moderate between group effect sizes against no treatment control conditions (6, 9) in trials on ICBT for adolescents, thus suggesting that treatment is better than no treatment which is in line with the literature on adults (10). Since youth anxiety is associated with youth depression, a transdiagnostic approach with an aim to treat both conditions with the same treatment manual is of interest. Transdiagnostic ICBT shows medium to large effect sizes for both anxiety and depression in adults (11), but has been less studied in adolescents. Further, understanding which active treatment components positively affect outcome may enhance our understanding of how and why treatment works and potentially support the development of more effective interventions (9, 12).

In recent years knowledge and learning have been identified as potentially important factors in psychotherapy, and this is an emerging area of research (13, 14). Evaluating what clients learn, know, and remember during and after treatment is of particular interest in psychotherapies based on traditional CBT and internet-based CBT (ICBT) (13, 14). Educational components constitute a fundamental part of CBT, and in particular in ICBT where psychoeducative texts and treatment rationale aiming to stimulate new adaptive learning experiences play a major role. Psychoeducation has the general aim to educate individuals about their condition and its treatment in ways that subsequently will help them deal with their situation by engaging in more adaptive cognitions and behaviors as well as preventing maladaptive ones (15, 16). In CBT manuals as well as in CBT self-help texts, psychoeducation is an integral part, alongside a range of other treatment components targeting explicit learning experiences (such as cognitive restructuring) and components targeting more implicit learning experiences for example during exposure (17). Psychoeducation and knowledge acquisition have also been acknowledged as important factors in the care of somatic problems when evaluating interventions for conditions such as cancer and schizophrenia (18), or when educating the general public about mental health issues (19) Surprisingly, the role of psychoeducation has not been much studied in CBT, with the exception of a few studies. For example, three studies have evaluated knowledge as an outcome in ICBT (20–22). Explicit knowledge about the specific condition and its CBT treatment was increased following treatment of adults with social phobia (20), adults with eating disorders (*d* = 1.12; (21)), and adolescents with depression (*d* = 1.25; (22)). While Andersson et al. (20) identified a small correlation between knowledge acquisition and treatment outcome on a secondary measure, Strandskov et al. (21) and Berg et al. (22) did not find any association between knowledge gain and symptom reduction. On the other hand Berg et al. (22) reported that knowledge at baseline had a negative impact on subsequent treatment outcome (*r* = -.38), suggesting that adolescents who initially score lower on a knowledge test tend to improve more with the intervention. Thus, knowledge is potentially an interesting construct when evaluating ICBT, and also appears to be a distinct phenomena that is not just another way to measure negative affect and/or symptoms. However, the psychometric properties of the knowledge tests used in the above-mentioned studies was not adequate (i.e., Cronbach’s alpha < .70), and it is yet unclear to what extent knowledge acquisition is a relevant factor for symptom reduction during treatment both as an outcome and as a predictor. Thus, knowledge in relation to treatment outcome warrants further research.

Another way to target the role of knowledge and learning is to manipulate knowledge and learning during treatment, and to study the effects of such manipulations on treatment outcome. Inspired by cognitive science and educational research, researchers have systematically included memory support in CBT and evaluated if these modifications affect memory, learning, and symptom outcome in depressed adults (23–26). The studies to date show small but promising results, indicating that strategies to improve memory and learning of treatment content are positively associated with remembering treatment content, and can also lead to symptom reduction and increased individual functioning. Memory and learning thus seems to be modifiable during treatment and affect treatment outcome. Gumport et al. (27) showed that while clients think about and apply the treatment content covered in therapy, half of the time their beliefs and understanding of treatment content is incorrect. This also highlights the need to study ways to improve learning in CBT, which arguably could be even more important in ICBT when clients get instructions on how to do for example exposure in the absence of a therapist in the room. To our knowledge, no study has yet evaluated the effects of incorporating memory or learning support in ICBT. This treatment format can be regarded as an optimal context for experimental manipulations and examination of the effects of memory support (13, 25), especially as it is possible to include larger samples in ICBT trials (10).

A related perspective that is relevant for internet interventions is research on the use of persuasive designs, mainly by means of technical solutions, for example by incorporating the possibility to monitor progress during a program, getting rewards or using avatars. The use of persuasive designs has been shown to increase adherence in internet interventions (28), but to our knowledge memory of treatment has not been investigated in that literature. In sum, evaluating and understanding the role of knowledge and learning is an important area of research in ICBT (14, 25).

In ICBT for youth populations there is a high risk of attrition and it is a challenge to enhance treatment completion (29, 30), even if treatment adherence often has been ambiguously defined and seldom reported in studies (9). However, in two of our previous trials on adolescents with depression we used scheduled weekly chat-sessions as support, and found that adherence was better than in previous trials without chat-sessions and that there were fewer drop-outs (31, 32). Thus adding scheduled chat-sessions could potentially influence treatment outcome. Few studies have evaluated ICBT with chat-sessions, despite its potential suitability as a way to support and guide younger persons during treatment (33). In the adult population, guided ICBT has often been found to be better than unguided ICBT (34) and guidance might be even more important in youth populations. For instance, Neil et al. (35) found that monitoring and support in a self-help ICBT school-program for adolescents substantially increased adherence and exercise completion, compared with an unguided community sample. However, there is a lack of research on what form and amount of guidance that is of importance for outcome in adolescents (9). In sum, studies are needed to experimentally evaluate the role of knowledge support and learning but also the role of scheduled chat-sessions.

In this study, we used a factorial design to evaluate learning support and chat-sessions as ways to enhance treatment outcome. In factorial designs, it is possible to evaluate independent factors relative importance on treatment outcome as well as potential interaction effects (36–38). In light of previous research our hypotheses were that adolescents would benefit from both learning support and chat-sessions during ICBT. We had no hypotheses regarding interaction effects between these two conditions. A further exploratory aim was to evaluate if knowledge gain was related to symptom reduction.

## Method

### Trial Design

**Figure 1:**
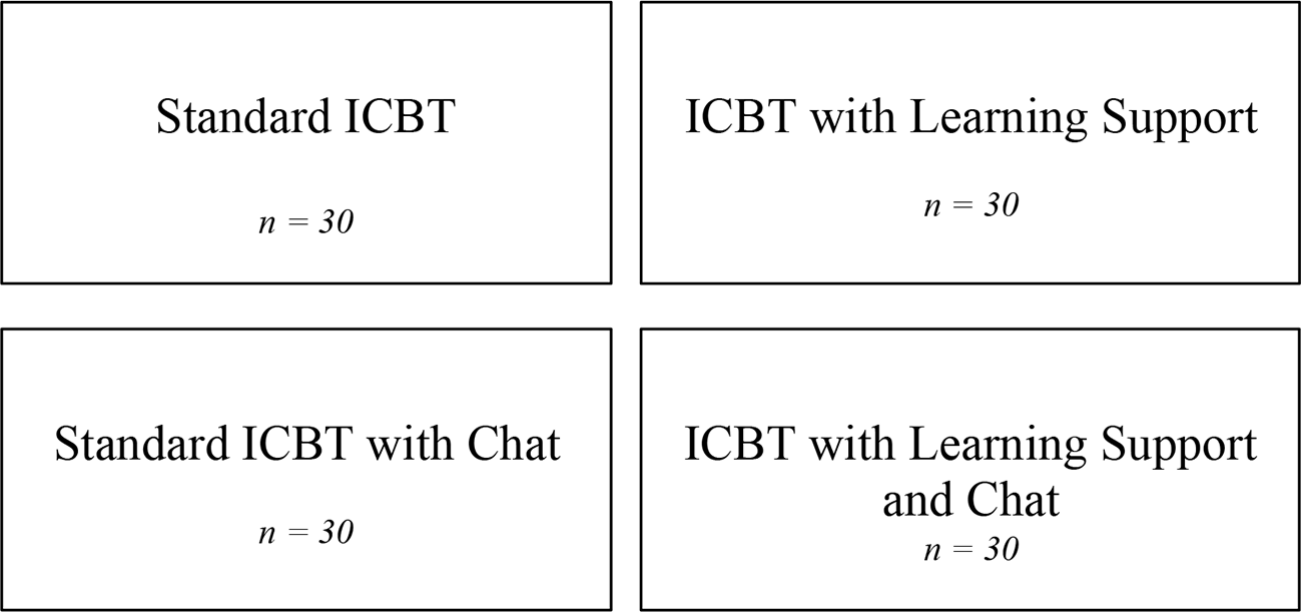
Illustration of the four conditions.

Following recruitment (see procedure), we included adolescents aged 15–19 years who suffered from anxiety and comorbid depressive symptoms. In order to examine the effects of two independent variables we randomized participants into one of four different treatment groups of ICBT using a full factorial design which allow estimates of main effects and interaction effects (39). The factors were a) with or without learning support incorporated into a standard therapist-guided ICBT (Factor 1) and b) with or without additional scheduled real time chat-sessions as an adjunct to asynchronous therapist-guided ICBT (Factor 2). The two factors thus had two levels (presence, coded as 1, absence coded as 0). The learning support consisted of strategies aimed at enhancing learning during treatment, and the participants either received a version of the treatment containing these strategies or a standard version of ICBT. Chat-sessions involved scheduled online chat-session with a therapist once a week, in addition to weekly feedback on the exercises. A quarter of the clients received none of the factors, a quarter received learning support but not chat, a quarter received chat but not learning support and a quarter received both learning support and chat. Thus, using a 2x2 factorial design, the participants (n = 120) received one of four different combinations of treatment modules and therapeutic support with 30 participants in each group. See **Figure 1** for overview.

Primary and secondary outcomes were measured at baseline, after 8 weeks post intervention and at 6 months follow up. Recruitment and assessment took place in January 2018, in Sweden, and treatments were delivered from February to April 2018. The follow-up was administered in October the same year.

All participants provided informed consent online. Ethics approval was granted by the region of Östergötland (reg.no 2017/489-31). The study was registered at ClinicalTRials.gov (NCT03441490).

### Participants

Participants were recruited from all over Sweden. To participate the adolescents were required to meet DSM-5 criteria for clinically relevant symptoms of anxiety, according to the Mini-International Neuropsychiatric Interview 7.0 for DSM-5 (M.I.N.I 7.0; (40, 41)), and/or score ≥ 7 points on the Beck Anxiety Inventory (BAI) if symptoms were reported in the interview but not enough to yield a diagnosis. All included patients were judged as suffering from clinically relevant anxiety, with our without presence of comorbid depressive symptoms. Further inclusion criteria were: if the participants were sufficient maturity to participate in research (based on a clinical impression), an age between 15 and 19 years, ability to read and write Swedish, have regular access to internet *via* a computer or smartphone, and for participants on medication (e.g., antidepressant) a stable dose for the past month. Exclusion criteria were ongoing psychological treatment interfering with the present study (e.g., CBT or a similar treatment), conditions of substance abuse (measured by Alcohol Use Disorder Identification Test and M.I.N.I 7.0.), eating disorders, acute suicidal ideation, psychosis, severe conditions of Attention deficit hyperactivity disorder (ADHD), or bipolar disorder, according to the M.I.N.I 7.0 (40). For AUDIT, we used the cut-offs ≥8 points for men, ≥6 points for women as guidelines. In Sweden, the age of consent for participating in a treatment study without parental consent is 15 years or older.

### Procedure

The study was mainly advertised in social media (e.g., Facebook and Snapchat), mental health care services, secondary schools and Swedish organizations for youth mental health. Interested individuals registered on the study’s home page, where they could read information about the purpose of the study, eligibility criteria, screening procedure, and the project groups.

For those who registered interest to participate in the study, screening procedures were carried out in two phases. First an email was sent containing instructions and a web link to the online assessment with the full range of outcome measures. Second, potentially eligible individuals were interviewed *via* telephone using the M.I.N.I 7.0 (40). Participants confirmed their identity by providing their personal identity number and full name in the interview. The diagnostic interviews were conducted by six psychology students in their final year of a 5-year program who were trained in using M.I.N.I 7.0 as a diagnostic tool. These same students were also the study therapists. They received supervision throughout the assessment and treatment procedure. The final decision about inclusion or exclusion was made by the principal investigator together with the psychology students and a licensed psychologist with experience of working with ICBT for adolescents.

**Figure 2:**
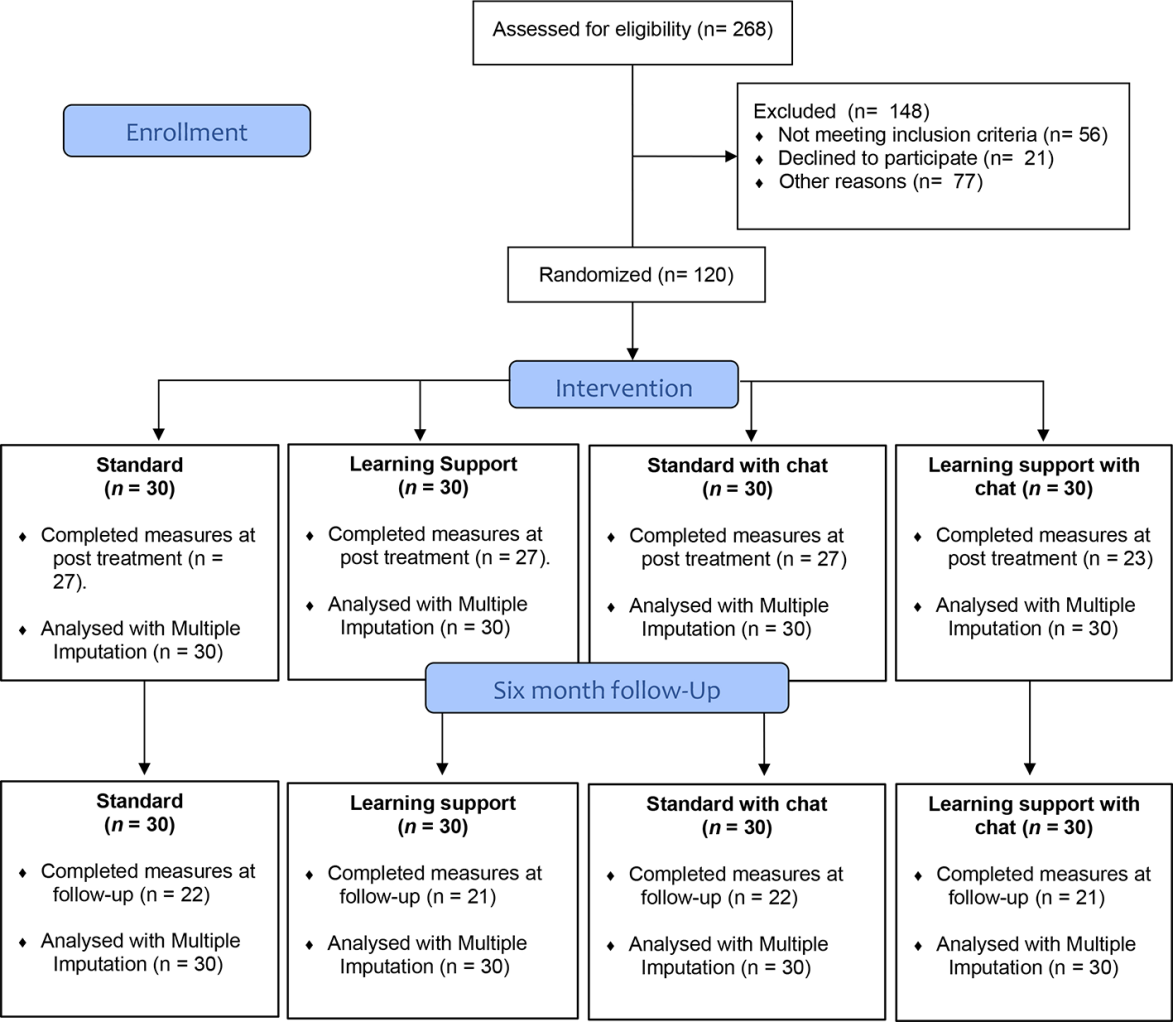
Flow chart of participants throughout the study.

Participants meeting the inclusion criteria were randomly assigned to one of the four treatment groups (1:1:1:1 ratio, as outlined previously). All of the included participants were informed about inclusion over the telephone and gave their informed consent verbally and later *via* the study platform before getting access to the modules. Adolescents below 18 years of age were not required to inform their parents/legal guardians about their participation in the study but were encouraged to inform an adult if possible. Participants were also informed that parents or legal guardians would be contacted in the event of severe clinical worsening, but not without informing the participant first. Excluded individuals were contacted *via* telephone and received a personal explanation about reasons for exclusion and guidance on how and where to seek suitable help. **Figure 2** presents a diagram of participant flow throughout the study.

### Randomization

Following screening procedures and inclusion the participants were randomized with no stratification. The randomization procedure was performed by an independent, off-site statistician, not involved in the study, using www.randomization.com.

### Clinical Assessment

All of the outcome measures were administered before and after treatment. The measure of Generalized Anxiety Disorder (GAD-7) was administered once a week throughout the treatment period. Since this was a transdiagnostic trial, a range of different outcome measures were used in order to capture and evaluate a wide range of symptoms. At pre-treatment, the full M.I.N.I.-7 (40) was administered *via* telephone to assess the presence of anxiety disorders, major depression disorder, and other comorbid diagnoses relevant for the study eligible criteria. M.I.N.I.-7 has good psychometric properties (40) and phone interviews are comparable to face-to-face assessment in terms of reliability (42). In order to lessen the burden for the adolescents, diagnostic assessments post-treatment were conducted using only M.I.N.I 7.0. We only assessed diagnoses that participants had fulfilled at baseline. During the post treatment telephone interview, the therapist also estimated the change in participants’ symptom severity from the baseline assessment, based on the Clinical Global Impression-Improvement Scales (CGI; (43)). CGI is a brief assessment tool rating clinical change on a graded scale ranging from 1 (Very much improved) to 7 (Very much worse).

#### Primary Outcomes

The primary outcome measure of clinical anxiety was the Beck Anxiety Inventory (BAI; (44)). BAI is a 21-item scale assessing physiological and cognitive symptoms of anxiety, scoring each item on a 0–3 point scale. Cut-off values are 8–15 for mild anxiety, 16–25 moderate anxiety, and 26–63 for severe anxiety (45). BAI has demonstrated high internal consistency and acceptable test-retest reliability. In the present sample, Cronbach’s alpha for the BAI was.90. BAI is indented for use from ≥ 17 years (45).

The primary outcome of comorbid depression was the Beck Depression Inventory-II, containing 21 items (BDI-II; (45). A total score between 14–19 points indicates mild depression, 20–28 points moderate depression, and 29–63 severe depression. BDI-II is the most valid and commonly used measure of adolescent depression (46), recommended use ≥ 13 years, and has high internal consistency and test-retest reliability (45). In the present sample, Cronbach’s alpha for the BDI-II was.89.

#### Secondary Outcomes

The Penn State Worry Questionnaire for Children (PSWQ-C; (47)) was used as secondary measure of general anxiety. Social phobia was assessed using Mini-Social Phobia Inventory (Mini-SPIN; (48)) and fearful cognitions were assessed using Agoraphobic Cognitions Questionnaire (ACQ; (49) Further, the Rosenberg Self-Esteem Scale (RSES; (50)) was used to assess individual self-esteem, and Satisfaction With Life Scale (SWLS; (51)) was used to measure global judgement of one’s life satisfaction. The AUDIT was in the baseline screening to assess individual drinking habits (52).

Finally, a brief 16-item questionnaire measuring explicit, declarative knowledge about core CBT-principles and therapeutic points in ICBT was used to evaluate knowledge gain during treatment. Example of item: *Molly is afraid of going downtown when the city is crowded with people. According to CBT, what could she try to do?* (Item 8). The knowledge test was developed by our research group (53). In the present study, the questionnaire was adapted to fit treatment content appropriately and to keep the number of items as low as possible. The adapted version of the knowledge test had an acceptable, close to high internal consistency with a Cronbach’s alpha of.79. Further, the test measured level of certainty on each item, using a 3-point Likert scale ranging from “I am guessing (0) to “I am very certain (2). Thus, the knowledge test was scored in two ways. First, we calculated a score based on the number of correct answers, ranging from 0 to 16 points, higher scores indicating higher knowledge. Second, we calculated a weighted score ranging between -16 to 32, using an established method (20–22), where the certainty-ratings were incorporated with the response to each item. If participants were correct and certain they received a higher score, if they were correct but guessing or uncertain they received a lower score, and if incorrect but certain they received a negative score. For a complete view of the knowledge test, see **Appendix B**.

#### Weekly Assessment

For the weekly assessment we used the measure Generalized Anxiety Disorder (GAD-7). GAD-7 is a measure with seven items that was administered weekly with the aim to monitor for anxiety symptom severity. Since GAD-7 is a brief measure and covers relevant aspects of anxiety such as “Feeling nervous, anxious or on edge” (Item 1) and covers one of the cardinal symptoms of depression for youths “Becoming easily annoyed or irritable” (Item 6) it was found suitable to use as the weekly measure in this trial. Scores between 0 and 4 are rated as minimal, 5 to 9 as mild, 10 to 14 as moderate and 15 to 21 as severe anxiety. The questions were re-formulated to include measure symptoms during the last week and not the last 2 weeks, as in the original version. GAD-7 has demonstrated high internal consistency and test-retest reliability for adults (54). In the present sample, Cronbach’s alpha for the GAD-7 was.83.

### Treatment

All participants received transdiagnostic therapist-guided ICBT delivered over 8 weeks. The intervention was delivered using a secure platform (55), with each participant having a password protected account requiring two-step authorization. Study therapists were randomly assigned approximately 20 participants each; five participants from each treatment group. The treatment was delivered in four different conditions.

#### Standard ICBT

Based on previous treatment modules developed by our group and used in trials for adolescents with anxiety and depression (31, 32, 56, 57), a new transdiagnostic treatment program was developed. The transdiagnostic treatment program contained eight modules, one per week, targeting a range of behavioral and cognitive components known to reduce anxiety and depression. Module 1 contained an introduction to the current treatment and CBT, as well as psychoeducation about anxiety and comorbid symptoms of depression. Module 2 focused on negative thoughts, how negative thoughts tend to generate negative emotions and how thoughts can be managed and challenged. Module 3 introduced how actions tend to affect our mood, how to increase valued behaviors and healthy sleep-routines. Module 4 focused on the differences between short and long-term consequences and how they affect our behavior by practicing functional analyses. Module 5 contained a rational and exercises for gradual exposure. Module 6 consisted of continued practice of exposure and psychoeducation about how to handle struggles or difficulties when trying to challenge ones fears. Module 7 contained information about different types of feelings and how to manage them, as well as a rational about how to increase self-esteem according to CBT. Module 8 focused on maintenance of new strategies and relapse prevention. For a more detailed description of the treatment modules, see **Appendix A**.

The 8-week transdiagnostic ICBT treatment modules without learning support was constructed without any formulations, interventions, and rationales encouraging active reflection, memorization, or application of treatment content, including video and image content. The purpose was to develop modules formulated as neutral and straightforward as possible, containing all relevant content and exercises used in a standard ICBT program but without pedagogical components woven into the texts. In this Standard ICBT condition, participants were given feedback and support from their therapist *via* mail through the study platform once a week. The feedback was tailored to each participant but had had an overall aim to motivate and help the participants engage with the exercises presented in the program.

#### ICBT With Learning Support

In this version of the ICBT treatment, learning support was incorporated into the standard transdiagnostic modules described above. The learning support involved strategies known to strengthen memory, deepen knowledge, and make information more accessible and durable in a long-term perspective (13, 58). In order to increase knowledge it is important to encode information so that it can be retrieved later when needed in difficult situations (59). Inspired by the cognitive support strategies used by Harvey et al. (13) and the work made by Bjork and Soderstrom (58) Hattie and Donoghue (59) we incorporated ways to mobilize attention to relevant information, stimulate continuous repetition, practice remembering, and actively process the material by applying, elaborating, and generalizing the content into various everyday situations. Important therapy points, principles and strategies of the treatment were decided a priori. The learning strategies were formulated and incorporated into the modules in several ways. For instance, short summaries, pedagogical pictures (60) and videos were included to highlight core therapy points and principles. Further, in each new module, the participants were given a quiz about the last module’s main therapy points and were asked to summarize the module in their own words in a text box. Main therapy points where applied on fictive cases and the participants were continuously encouraged to actively reflect upon the content in the modules through questions asking about how the current content related to what they already knew or had tried, how they would apply it in their own life, or how they would describe the content if talking to a friend. See Module overview and learning support strategies in **Appendix A**.

#### Standard ICBT With Chat-Sessions

This group received the standard version of the ICBT program without incorporated learning support. In addition to receiving guidance through email on a weekly basis, participants were also invited to chat with their therapist each week, in the form of scheduled 30 min chat sessions. Chat-sessions were task-focused, aiming to motivate and help participants to perform exercises, and to support participants that in some way struggled with the treatment content.

#### ICBT With Learning Support and Chat-Session

This treatment condition included both learning support and scheduled chat-sessions (see description above).

### Statistical Analyses

Statistical analyses were performed using SPSS version 24. We used the software g*power to estimate sample size (61). Based on previous effect sizes in ICBT treatment studies on adolescents (6, 9) we estimated an sample of 120 participants (30 in each condition, with a total of 60 for each contrast) in order to obtain 80% power with a two-sided alpha level of.05 and an effect size equivalent to an end-point difference of *d* = 0.52. The presentation of the results is divided by Complete Case Analysis and Intention-to-treat analyses (ITT). The treatment effects for all of the primary and secondary outcomes of completers-only and the ITT datasets were analyzed using analysis of covariance (ANCOVA). In line with recommendations from Van Breukelen (62) and Vickers and Altman (63), we used baseline values as covariates. We used multiple imputation (MI) to account for missing data at post-treatment and at 6-month follow-up. The use of MI relies on the assumption that data is Missing at Random (MAR), i.e., allows that the probability of missing data on a variable to be dependent on any observed variable, but not to the would-be values of the missing data point (64). In principle, it is impossible to test whether the assumption of MAR holds, but since we did not find any pattern in the missing data with regards to sociodemographic variables and symptom levels at baseline, MAR was a justifiable assumption. The parameter estimates were pooled from 10 sets of imputed data. We also used general mixed models with unstructured equation modelling to calculate the treatment effects on the weekly measures of GAD-7. Mixed models also rely on the assumption that missing data is MAR. Within and between-group effect sizes were reported using Cohen’s *d* and corresponding 95% CI, where the differences in means between pre- and post-treatment was divided by the pooled standard deviation. According to Cohen (65), *d* = 0.20 can be considered a small effect, *d* = 0.50 a medium and *d* = 0.80 a large effect. In order to assess correlations between treatment outcomes and knowledge scores, the Pearson correlation coefficient was used.

#### Improvement and Seterioration

Improvement was defined by using both clinically relevant change and the reliable change index (66). Clinical significant change was calculated to evaluate if clients were more likely to be closer to the mean of the functional population than the mean of the dysfunctional population, at the end of treatment (66). In this study this was defined as having a score on the BDI-II and the BAI at post treatment within one standard deviation of the mean in a nonclinical population. This resulted in a cut-off point of 19.87 (in the functional population: *M* = 10.75, *SD* = 9.12) for the BAI using student population norm data from Borden et al. (67), and 23.00 (in the functional population: *M* = 10.75, *SD* = 10.50) for the BDI-II using youth population norm data from Osman et al. (68). Reliable change index was calculated to investigate the number of participants changing significantly and not due to measurement error, where change scores should exceed 1.96 times the SD of the measurement (66). In the present sample, this meant that a participant had to have a change score of > 8.76 on the BAI and a score of > 12.55 on the BDI-II in order to have changed reliably.

Deterioration was defined by using a negative change score exceeding the reliable change index on BAI and BDI, as recommended by Jacobson & Truax (66). Occurrence of negative effects during the treatment period were assessed with open-ended questions, in accordance with consensus statement on negative effects in ICBT (69).

As an additional measure of improvement and deterioration we used the CGI-scores obtained at the post treatment phone interview. A score of 3 and below was categorized as improvement, a score of 4 as unchanged and scores of 5 or higher as deterioration (43). For both improvement and deterioration, we analyzed the Complete cases. Missing cases were categorized as unchanged.

## Results

### Sample Characteristics

The included participants were mostly female (81%), with a mean age of 17 years (*SD* = 1.20) and who went to school (91%). **Table 1** presents baseline characteristics for the whole sample. The four groups did not differ significantly on any continuous baseline variable (all *p’*s > .05), or demographic characteristics, with two exceptions. The group that received Standard ICBT with Chat-sessions had received more previous treatments than participants in other groups [χ^2^(3) = 7.92, *p* =.05], and in the group that received ICBT with Learning support and Chat-sessions the prevalence of ADHD was higher compared to other groups [χ^2^(6) = 14.03, *p* =.03]. We did not assume that these two factors would have an influence on the outcome but checked this assumption later.

**Table 1:** Baseline characteristics of the participants in the study.

Demographics	Standard ICBT *n = 30*	Learning Support *n = 30*	Standard with Chat *n = 30*	Learning support with Chat *n = 30*	Totalt *n = 120*
**Age**, M (SD)	16.97 (1.19)	17.20 (1.16)	16.97 (1.13)	16.73 (1.34)	16.97 (1.20)
**Gender**, n (%)					
*Girl*	24 (80.0)	24 (80.0)	27 (90.0)	22 (73.3)	97 (80.8)
*Boy*	6 (20.0)	6 (20.0)	3 (10.0)	7 (23.3)	22 (18.3)
*Other*	0 (0.0)	0 (0.0)	0 (0.0)	1 (3.3)	1 (0.8)
**Residence**, n (%)					
*City*	14 (46.7)	8 (26.7)	14 (46.7)	8 (26.7)	44 (36.7)
*Small Town*	11 (36.7)	12 (40.0)	11 (36.7)	15 (50.0)	49 (40.8)
*Rural area*	5 (17.2)	10 (33.3)	5 (16.7)	7 (23.3)	27 (22.5)
**Occupation**, n (%)					
*School*	26 (86.7)	29 (96.7)	29 (96.7)	26 (86.7)	110 (91)
*Work*	2 (6.7)	1 (3.3)	0 (0.0)	3 (10)	6 (5.0)
*Other*	0 (0.0)	0 (0.0)	0 (0.0)	1 (3.3)	1 (0.8)
**Previous mental health contact**, n (%)					
*None*	25 (83.3)	24 (80.0)	16 (53.3)	21 (70.0)	86 (71.7)
*Yes, earlier*	3 (10.0)	6 (20.0)	11 (36.7)	6 (20.0)	24 (20.0)
*Yes, right now*	3 (10.0)	4 (13.3)	3 (10.0)	4 (13.3)	14 (11.7)
*Psychotropic medication*	5 (16.7)	5 (16.7)	8 (26.6)	5 (16.7)	23 (19.2)
**Anxiety Disorders**¹,					
*Social Anxiety*	15 (50.0)	15 (50.0)	16 (53.3)	17 (56.7)	63 (52.5)
*General Anxiety*	12 (40.0)	13 (43.3)	11 (36.7)	13 (43.3)	49 (40.8)
*Disorder*					
*Panic disorder*	10 (33.3)	9 (30.0)	14 (46.7)	7 (23.3)	40 (33.3)
*Agoraphobia*	7 (23.3)	4 (13.3)	8 (26.7)	5 (16.7)	24 (20.0)
*Obsessive*					
*Compulsive disorder*	4 (13.3)	5 (16.7)	3 (10.0)	5 (16.7)	17 (14.2)
**Major Depression** ^2,^	18 (60.0)	18 (60.0)	18 (60.0)	17 (56.7)	71 (59.2)
**ADHD** ^3^	2 (6.7)	1 (3.3)	2 (6.7)	7 (23.3)	12 (10.0)

^1,2,3^Confirmed by M.I.N.I-7.

### Treatment Dropout and Missing Data

Of the total sample (*n* = 120), 104 (87%) completed all outcome measures at post treatment. However, two additional participants completed only the BAI, leading to a total *n* of 106 (88%) completing the primary outcome on anxiety. A total of 98 (82%) participants completed the diagnostic telephone interview at post treatment. Using *t*-tests and χ^2^-tests, no differences on the baseline outcome measures were detected between completers and non-completers in the four conditions, all *p’*s > .05. Little MCAR’s test was non-significant, χ^2^(5) = 2.00, *p* =.85, indicating that there was no obvious pattern explaining missing data.

Of the total sample of 120 participants, 15 (12.5%) dropped out of treatment, i.e., expressed that they no longer wanted to participate in the study. All drop-outs filled out the assessment at post treatment and were included in analyses according to the intention-to-treat principle. See Flow chart.

Follow-up data on the primary outcomes at 6 months follow-up were obtained from 88 participants of the original sample (73%). A total of 82 participants (66%) completed all follow-up measures including the knowledge test. At follow up, BAI, BDI-II, SWLS, RSES, and the knowledge test were administered, thus excluding the three secondary outcomes of anxiety, PSWQ, ACQ, and MINI-SPIN. This was done to reduce the burden for the participants to answer questionnaires.

### Treatment Adherence

On average, each participant completed 5.46 (64.0%) out of eight modules (*SD* = 2.82), defined as opening a module and completing at least one exercise associated per module. Of all 120 participants, 47 (39.2%) completed all assigned modules. A total of 14 participants logged in and answered messages from their therapist but did not complete any of the exercises, thus were categorized as completing zero modules. A one-way ANOVA showed no differences between the four treatment groups regarding compliance as measured by the number of completed modules, *F*(3, 115) = 0.99, *p* =.40. Furthermore, participants in the chat condition participated on average in 5.68 (71.0%) eight chat-sessions (*SD* = 3.01). Of all participants in the two chat conditions, 60 (48.3%) participated in all eight chat-sessions. The *t*-test showed no differences between the groups that received chat-sessions in the number of sessions completed, *t*(58) = 1.25, *p* =.22.

### Overall Results on Primary Outcomes

Descriptive statistics for each of the conditions at each assessment point is presented in **Table 2**. Imputed means are presented in Appendix C. Within-group and between-group effect sizes for BAI, BDI, and knowledge scores (Cohen’s *d*) are presented in **Table 3**. The within-group and between-group effect sizes for all of the secondary outcomes are presented in Appendix D.

**Table 2:** Estimated means, standard deviations, and ns for each outcome measure divided by condition and assessment point*.

Measure and condition	Pre			Post (10 weeks)			Follow-up (6 months)		
	*M*	*SD*	*N*	*M*	*SD*	*N*	*M*	*SD*	*N*
**Beck Anxiety Inventory**									
Standard ICBT	26.57	12.25	30	19.71	10.85	28	15.32	11.01	22
Learning support	22.22	11.16	30	11.70	8.74	27	11.14	7.20	21
Standard ICBT with chat	28.89	12.62	30	21.14	12.03	28	15.64	12.59	22
Learning support with chat	28.44	9.67	30	18.87	10.83	23	16.06	14.20	21
**Beck Depression Inventory**									
Standard ICBT	29.26	10.12	30	19.56	14.91	27	16.43	13.32	22
Learning support	25.37	10.08	30	11.70	8.20	27	9.86	8.97	21
Standard ICBT with chat	27.70	12.15	30	16.96	15.70	27	12.95	15.97	22
Learning support with chat	30.70	9.51	30	15.70	9.78	23	12.16	12.02	21
**Penn State Worry Questionnaire for Children**									
Standard ICBT	27.74	7.33	30	26.07	7.86	27	Na*	na	na
Learning support	27.89	8.01	30	25.15	9.13	27	na	na	na
Standard ICBT with chat	29.11	7.64	30	24.52	8.43	27	na	na	na
Learning support with chat	29.70	5.53	30	26.00	7.77	23	na	na	na
**Mini-Social Phobia Inventory**									
Standard ICBT	6.11	3.48	30	4.85	3.62	27	na	na	na
Learning support	5.78	3.75	30	4.78	3.75	27	na	na	na
Standard ICBT with chat	6.52	4.34	30	5.63	3.96	27	na	na	na
Learning support with chat	7.52	3.70	30	5.70	3.01	23	na	na	na
**Agoraphobic Cognitions Questionnaire**									
Standard ICBT	2.06	065	30	1.80	0.56	27	na	na	na
Learning support	1.70	0.56	30	1.48	0.62	27	na	na	na
Standard ICBT with chat	2.11	0.71	30	1.93	0.79	27	na	na	na
Learning support with chat	2.07	0.56	30	1.78	0.59	23	na	na	na
**Satisfaction With Life Scale**									
Standard ICBT	13.00	5.43	30	14.96	5.78	27	17.29	4.67	22
Learning support	15.37	4.07	30	17.41	4.52	27	17.48	5.72	21
Standard ICBT with chat	14.96	5.37	30	15.52	5.91	27	16.80	6.22	21
Learning support with chat	13.52	4.56	30	15.70	5.80	23	18.00	6.07	21
**Rosenberg Self-Esteem Scale**									
Standard ICBT	11.70	5.31	30	12.96	6.30	27	17.24	5.19	22
Learning support	14.22	3.97	30	16.26	4.36	27	19.24	6.42	21
Standard ICBT with chat	12.37	6.91	30	15.74	7.79	27	18.10	7.55	21
Learning support with chat	12.39	5.72	30	16.22	6.42	23	17.74	8.43	21
**Knowledge test, raw scores**									
Standard ICBT	12.57	2.38	30	14.50	1.75	28	13.90	3.12	21
Learning support	11.74	2.68	29	14.48	2.08	27	14.05	2.90	21
Standard ICBT with chat	12.11	3.26	30	13.93	2.39	28	14.70	2.13	20
Learning support with chat	11.50	3.61	29	14.71	2.40	23	14.32	2.83	19
**Knowledge test, weighted scores**									
Standard ICBT	14.32	3.96	30	22.00	7.34	28	19.44	7.10	21
Learning support	13.74	4.59	29	23.59	6.75	27	21.14	8.10	21
Standard ICBT with chat	14.36	5.93	30	21.86	7.21	28	23.65	7.05	20
Learning support with chat	12.71	4.29	29	24.04	6.54	23	24.42	7.43	19

*Observed means based on completers. Na; Not applicable, measures not administered at follow-up.

**Table 3:** Within-group effect sizes and between-group effect sizes, presented as Cohen’s *d* [95% CI] for primary outcomes and knowledge at each assessment point.

	BAI	Measure		Knowledge test, Weighted score
		BDI	Knowledge test Raw score	
**Between-group effect sizes pre to post treatment**				
Learning support vs no learning support				
*Completers*	0.41 [0.05, 0.77]	0.37 [0.01, 0.73]	0.46 [0.09, 0.82]	0.49 [0.12, 0.85]
*ITT*	0.38 [0.01, 0.73]	0.34 [-0.02, 0.70]	0.38 [0.01, 0.74]	0.42 [0.06, 0.78]
Chat-sessions vs no chat-sessions				
*Completers*	0.24 [-0.60, 0.12]	0.03 [-0.33, 0.39]	0.12 [-0.48, 0.24]	0.10 [-0.25, 0.46]
*ITT*	0.23 [-0.59, 0.13]	0.04 [-0.32, 0.39]	0.02 [-0.38, 0.36]	0.02 [-0.34, 0.38]
**Within-group effect sizes pre to post treatment**				
Standard ICBT				
*Completers*	0.59 [0.06, 1.11]	0.77 [0.22, 1.30]	0.92 [0.37,1.45]	1.32 [0.73, 1.86]
*ITT*	0.63 [0.10, 1.13]	0.75 [0.22-1.26]	0.80 [0.27, 1.32]	1.34 [0.77, 1.88]
Learning support				
*Completers*	1.04 [0.48, 1.58]	1.48 [0.87, 2.04]	1.14 [0.56, 1.68]	1.72 [1.08, 2.31]
*ITT*	0.98 [0.44, 1.51]	1.33 [0.75, 1.87]	1.11 [0.55, 1.64]	1.68 [1.07, 2.25]
Standard ICBT with chat				
*Completers*	0.63 [0.09, 1.15]	0.77 [0.22, 1.30]	0.63 [0.10, 1.15]	1.14 [0.57-1.69]
*ITT*	0.66 [0.13, 1.17]	0.79 [0.26, 1.31]	0.66 [0.13, 1.17]	1.16 [0.60, 1.69]
Learning support with chat				
*Completers*	0.94 [0.35, 1.50]	1.56 [0.92, 2.15]	1.02 [0.43, 1.59]	2.10 [1.39, 2.74]
*ITT*	0.85 [0.21, 1.36]	1.39 [0.81, 1.94]	1.02 [0.47, 1.55]	1.88 [1.25, 2.47]
**Between-group effect sizes pre to 6 month follow-up**				
Learning support vs no learning support				
*Completers*	-0.14 [-0,56, 0.62]	-0.29 [-0.71, 0.14]	-0.05 [-0.48, 0.37]	0.01 [-0.43, 0.42]
*ITT*	-0.09 [-0.27, 0.44]	-0.20 [-0.56, 0.16]	-0.07 [-0.43, 0.29]	0.01 [-0.37, 0.34]
Chat-sessions vs no chat-sessions				
*Completers*	0.20 [-0.23, 0.62]	0.01 [-0.41, 0.44]	0.14 [-0.29, 0.56]	0.10 [-0.25, 0.46]
*ITT*	0.14 [-0.22, 0.50]	0.02 [-0.33, 0.38	0.12 [-0.24, 0.48]	0.02 [-0.34, 0.38]
**Within-group effect sizes post treatment to 6 months follow-up**				
Standard ICBT				
*Completers*	0.40 [-0.17, 0.96]	0.21 [-0.36, 0.77]	0.25 [-0.32, 0.81]	0.35 [-0.22, 0.92]
*ITT*	0.38 [-0.14, 0.88]	0.26 [-0.76, 0.25]	0.29 [-0.22, 0.80]	0.00 [-0.51, 0.51]
Learning support				
*Completers*	0.07 [-0.50, 0.64]	0.22 [-0.36, 0.79]	0.17 [-0.40, 0.74]	0.33 [-0.25, 0.90]
*ITT*	0.09 [-0.41, 0.60]	0.03 [-0.54, 0.47]	0.21 [-0.30, 0.74]	0.28 [-0.23, 0.79]
Standard ICBT with chat				
*Completers*	0.45 [-0.12, 0.01]	0.30 [-0.27, 0.87]	0.34 [-0.25, 0.91]	0.26 [-0.84, 0.32]
*ITT*	0.33 [-0.83, 0.18]	0.16 [-0.35, 0.67]	0.10 [-0.41, 0.61]	0.20 [-0.31, 0.71]
Learning support with chat				
*Completers*	0.22 [-0.35, 0.78]	0.32 [-0.28, 0.91]	0.15 [-0.76, 0.46]	0.00 [-0.61, 0.61]
*ITT*	0.19 [-0.69, 0.32]	0.22 [-0.29, 0.73]	0.14 [-0.37, 0.64]	0.05 [-0.45, 0.56]

BAI, Beck Anxiety Inventory; BDI, Beck Depression Inventory.

At post-treatment, a medium sized within-group treatment effect was found on the BAI, *t*(104) = 7.71, *p* < .001, *d* = 0.75, 95% CI [0.47, 1.03], and a large treatment effect on the BDI-II, *t*(102) = 11.06, *p* < .001, *d* = 1.04, 95% CI [0.76, 1.32], showing an overall decrease in symptoms of anxiety and depression for all of the participants independent of treatment condition.

When evaluating the effects of the two independent variables on the BAI at post treatment, the results from the ANCOVA revealed a main effect of learning support, *F*(1, 101) = 4.86, *p* =.03, *d* = 0.41, 95% CI [0.05, 0.77], with lower scores for the learning support conditions. There was no main effect of receiving chat-sessions as part of treatment, *F*(1, 100) = 1.72, *p* =.19. Further, no interaction effects were found between learning support and chat-sessions, *F*(1, 100) = 1.20, *p* =.28. For the BDI-II, the ANCOVA revealed a main effect of learning support, *F*(1, 99) = 4.15, *p* =.04, *d* = 0.37, 95% CI [0.01, 0.73] with lower post-treatment depression scores for the learning support conditions. No effect of chat-sessions, *F*(1, 99) = 0.07, *p* =.80, or any interaction effect between the two factors *F*(1, 99) = 0.23, *p* =.63, were detected. Results remained similar when controlling for a probable diagnosis of ADHD.

### Secondary Outcomes

There were small effects on the secondary outcomes of PSWQ, ACQ, MINI-SPIN, RSES, and SWLS, *t*(102) = 4.17–5.67, all *p’*s < .001, *d’*s = 0.30–0.46, 95% CI [0.04, 0.73], showing lower scores of anxiety and higher scores of self-esteem and quality of life for all of the participants independent of treatment condition. The results also revealed a large treatment effect on knowledge gain for the raw knowledge scores, *t*(104) = 11.38, *p* > .001, *d* = 0.91, 95% CI [0.63, 1.18], and a large treatment effect on the weighted knowledge scores *t*(104) = 16.31, *p* > .001, *d* = 1.53, 95% CI [1.23, 1.82].

ANCOVAs were also conducted for the PSWQ, MINI-SPIN, ACQ, SWLS, and RSES post scores, controlling for pre-treatment scores. No main effects of learning support or chat-sessions could be detected and no interaction effects between the two factors *F*(1, 99) = 0.00-1.36, all *p*s > .5. One exception, however, was a main effect of chat-session on self-esteem (RSES), *F*(1, 99) = 4.34, *p* =.04, with higher self-esteem scores for the chat-session conditions compared to the other conditions. No effect was found on RSES of learning support, *F*(1, 99) = 9.43, *p* =.33, and no interaction effects between chat-sessions and learning support on RSES, *F*(1, 99) = 0.21, *p* =.65.

#### Knowledge Test

The ANCOVAs conducted on knowledge acquisition showed a main effect of learning support, both for the total scores of correct answers, i.e., raw knowledge scores, *F*(1, 101) = 6.13, *p* =.02, *d* = 0.46, 95% CI [0.09, 0.82] as well as for weighted scores including level of certainty, *F*(1, 101) = 7.04, *p* =.01, *d* = 0.49, 95% CI [0.12, 0.85]. Thus, participants receiving learning support had higher scores of knowledge at post treatment than the other group who had not received learning support. No independent main effect of chat-sessions could be found for raw knowledge scores, *F*(1, 101) = 0.001, *p* =.98, or weighted scores, *F*(1, 101) = 0.31, *p* =.58. Further, no interaction effects between learning support and chat-sessions were found on knowledge gain on either of the knowledge scores, *F*(1, 101) = 1.13, *p* =.26, *F*(1, 101) = 0.51, *p* =.48.

Finally, no association between knowledge gain and symptom reduction during treatment could be found either for anxiety (BAI; raw knowledge scores; *r* = -.05, *p* =.61; weighted scores; *r* = -.07, *p* =.51) or for depression (BDI-II; raw knowledge scores*; r* = -.01, *p* =.96; weighted scores; *r* = -.13, *p =.*21). Further, pre-treatment knowledge levels did not predict treatment outcome on either the BAI (raw knowledge scores; *r* = -.07, *p* =.50; weighted scores; *r* = -.07, *p* =.45), or on the BDI-II (raw knowledge scores; *r* = -.07, *p* =.46; weighted scores; *r* = -.16, *p* =.12).

### Intention-to-Treat-Analysis

We repeated the calculations performing ITT analyses with imputed missing data. There was an medium treatment effect on the main outcomes of BAI, *t*(118) = 7.39, *p* < .001, *d* = 0.72, 95% CI [0.45, 0.98] and a large effect on BDI, *t*(118) = 10.41, *p < .*001, *d* = 0.97, 95% CI [0.70, 1.23].

Using the imputed data set, an ANCOVA revealed a main effect of learning support on the primary anxiety measure BAI, *F*(1, 115) = 3.99, *p* =.05, *d* = 0.38, 95% CI [0.01, 0.73]. On the depression measure BDI, the ANCOVA showed a trend towards a main effect of learning support, *F*(1, 115) = 3.60, *p* =.06, *d* = 0.34, 95% CI [-0.02, 0.70]. As in the Complete Case analyses, no main effects of receiving chat-sessions and no interaction effects between learning support and chat-sessions were found on either BAI or BDI, *F*(1, 115) = 0.13-2.28, all *p*s > .05. Results remained similar when controlling for ADHD.

On the secondary outcomes, results were essentially the same as for the Complete Case Analysis. There were small treatment effects on all the secondary outcomes PSWQ, ACQ, MINI-SPIN, RSES, and SWLS *t*(118) = 3.83-5.48, all *ps* > .001, *d* = 0.31–0.41, 95% CI [0.09, 0.67], revealing lower scores of anxiety and higher scores of self-esteem and quality of life for all of the participants independent of treatment condition. Further, there was a large treatment effect on knowledge gain for raw scores, *t*(116) = 11.66, *p* > .001, *d* = 0.91, 95% CI [0.64, 1.18], and a large treatment effect on weighted knowledge scores *t*(116) = 16.49, *p* > .001, *d* = 1.50, 95% CI [1.21, 1.78]. Thus, the treatment increased knowledge levels.

No effects of either learning support, chat-sessions or interaction effects could be found on either PSWQ, MINI-SPIN, ACQ, SWLS, or RSES, *F*(1, 115) = 0.14–2.62, all *p*s > .05.

The ANCOVAs using imputed data showed that learning support had a main effect on both the raw knowledge scores, *F*(1, 115) = 4.21, *p* =.04, and the weighted scores, *F*(1, 115) = 5.36, *p* =.02, with higher scores for the participants receiving learning support. The ANCOVAs showed no effects for chat-sessions on knowledge gain and did not revealed any interaction effects, *F*(1, 115) = 0.10-0.62, all *p*s > .05.

Finally, no association between knowledge gain and symptom reduction during treatment could be found on either the BAI (raw knowledge scores; *r* = -.05, *p* =.61; weighted scores; *r* = -.07, *p* =.51) or the BDI-II(raw knowledge scores*; r* =.-01, *p* =.96; weighted scores; *r* = -.13, *p =.*21). Further, knowledge levels pre-treatment did not predict treatment outcome on either the BAI (raw knowledge scores; *r* = -.06, *p* =.54; weighted scores; *r* =.-07, *p* =.51) or on the BDI-II(raw knowledge scores*; r* =.-06, *p* =.51; weighted scores; *r* = -.13, *p =.*21).

### Weekly Measures

Using mixed models, a significant effect was found on learning support by time -0.32, 95% CI [-0.57, -0.06], *t*(120) = -2,48, *p* =.02, showing that participants receiving learning support improved on average 0.32 points more on the GAD-7 per week, compared to participant for whom learning support was not part of treatment. No significant effects were found on chat-sessions by time, *t*(120) = -1,81, *p* =.07, and no interaction effects between learning support and chat-sessions was observed, *t*(120) = 1.69, *p* =.10.

### Improvement and Deterioration

A total of 67 (55.8%) participants reached the cut-off for clinically relevant change on the BAI and 80 (66.7%) on the BDI. A total of 47 (39.2%) participants had a reliable change according to the RCI on the BAI and 80 (66.7%) on the BDI.

Improvement on the BAI and BDI-II was defined as reaching the cut-off for clinically relevant change (19.87 for the BAI, 23.00 for the BDI), while also having reliable change according to the RCI. With missing cases defined as unchanged, a total of 34 participants (28.3%) improved on the BAI between pre and post treatment assessment and 26 participants (21.7%) improved between post assessment and 6-month follow-up. A total of 43 participants (35.8%) improved on the BDI-II during treatment and 21 (17.5%) improved during the 6 months period after ending treatment. See **Table 4** for rates of improvement and deterioration for each group. The χ^2^-tests showed no differences between the groups regarding the number of clinically improved participants or reliably deteriorated participants presented below, on either BAI or BDI, χ^2^(3) = 0.21–4.33, all *p’*s > .05.

**Table 4:** Rates of improvement and deterioration.

	Improvement		Deterioration	
	Pre to post (8 weeks)	Post to Follow-up (6 months)	Pre to Post (8 weeks)	Post to Follow-up (6 months)
Standard ICBT, n (%)				
*BAI,*	7 (23.3%)	7 (23.3%)	1 (3.3%)	4 (13.3%)
*BDI*	7 (23.3%)	5 (16.7%)	0 (0.0%)	0 (0.0%)
CGI*	21 (70.0%)	15 (50%)	1 (3.3%)	1 (3.3%)
Learning support, n (%)				
*BAI*	11 (36.7%)	3 (10.0%)	0 (0.0%)	4 (13.3%)
*BDI*	14 (46.7%)	2 (6.7%)	0 (0.0%)	0 (0.0%)
*CGI*	20 (66.7%)	7 (23.3%)	0 (0.0%)	1 (3.33%)
Standard ICBT with chat, n (%)				
*BAI*	7 (23.3%)	8 (26.7%)	1 (3.3%)	4 (13.3%)
*BDI*	10 (33.3%)	8 (26.7%)	0 (0.0%)	0 (0.0%)
*CGI*	24 (80.0%)	12 (40.0%)	0 (0.0%)	4 (13.3%)
Learning support with chat, n (%)				
*BAI*	9 (30.0%)	8 (26.7%)	2 (6.7%)	5 (16.7%)
*BDI*	12 (40.0%)	6 (20.0%)	0 (0.0%)	0 (0.0%)
*CGI*	23 (76.7%)	10 (33.0%)	0 (0.0%)	1 (3.33%)

BAI, Beck Anxiety Inventory; BDI, Beck Depression Inventory; CGI, Clinical Global Impression.

*CGI scores; 1–3, improved; 4, unchanged; 5–7, deteriorated.

Deterioration on the BAI and the BDI-II was determined by a reliable change score in the negative direction. On the BAI, a total of four participants (3.3%) deteriorated during the treatment period, and between post treatment assessment to the 6-month follow-up, 17 participants (14.2%) deteriorated. On the BDI-II, no client showed deterioration during treatment or at the 6-month follow-up. Further, open-ended questions about negative experiences related to treatment showed that six participants (5%) reported occasional feelings of getting worse while taking part of the treatment, or reported stress due to work-overload combined with a lack of self-efficacy. There were no statistical differences between the four groups in terms of reported negative effects, χ^2^(3) = 3.51, *p* =.32.

CGI was rated at the end of treatment and at 6-month follow-up. Participants rated with scores 1–3 were defined as improved, scores 5–7 as deteriorated and 4 as unchanged. See **Table 4** for rates about CGI in each treatment group.

Along the same lines, remission rates for other diagnoses present at baseline was 65.1% for Social Anxiety Disorder, 62.5% for Panic Disorder, 58.3% for Agoraphobia, 70.6% for Obsessive Compulsive Disorder, and 81.7% for Major Depression. There was no difference in the amount of diagnoses that participant no longer fulfilled criteria for in the four different groups, χ^2^(5) = 2.83–5.92, all *p’*s > .05.

### Therapist Time

The average time a therapist spent on each participant per week was 23.3 min (*SD* = 16.17). The minimum average time for one of the participants was 0 min and the highest one was 52.5 min. A one-way ANOVA, using *post hoc* analyses with Bonferroni-correction showed that a higher amount of treatment time for the two groups that received chat-sessions compared with those who did not receive chat-sessions, *F*(3, 115) = 3.24, *p* < .001. On average, the therapists spent 34.07 min per week on clients receiving chat-sessions as part of their treatment, compared to 12.61 min per week for participants that received treatment without chat-sessions.

### Six-Month Follow-Up

As predicted, there was a large effect of treatment 6 months later (pre to follow-up), on the two primary outcomes BAI and BDI-II, *t*(84) = 7.30, *p < .*001, *d* = 1.04, 95% CI [0.72, 1.35] and *t*(84) = 9.2, *p* < .001, *d* = 1.28, 95% CI [0.94, 1.60] respectively. Further, there was a medium respectively large treatment effect on the secondary outcomes of RSES and SWLS *t*(79) = 7.19–4.01, *p* < .001, *d* = 0.93–0.59, 95% CI [0.28, 1.24] and a medium respectively large effect of the raw and weighted knowledge scores, *t*(79) = 6.2–10.92, *p* < .001, *d* = 0.72, 95% CI [0.51, 0.1.88]. See **Table 4**.

Using paired samples *t*-test, results revealed that the outcomes on all of the measures were stable over time, *t*(80-75) = 0.28–1.70, all *p*s > .50. One exception was an increase on RSES over time, *t*(79) = 3.46, *p* < .001, thus showing that levels of self-esteem increased during the 6 months after ending treatment.

The analyses using ANCOVAs showed that there were no difference between the four treatment groups 6 months after treatment, *F*(1, 76-81) = 0.00–1.86, all *ps* > .50

Results remained similar in the ITT analyses, but with somewhat lower effect sizes. The treatments effects at 6-months were medium on the BAI and close to large on the BDI, *t*(118) = 7.57, *p* < .001, *d* = 0.92, 95% CI [0.65, 1.18] and *t*(118) = 9.96, *p* < .001, *d* = 1.17, 95% CI [0.89, 1.44] respectively. Further, medium treatment effect were observed for the secondary outcomes of RSES and SWLS *t*(118) = 7.43-4.48, *p* < .001, *d* = 0.85-0.57, 95% CI [0.31, 1.11]. A medium respectively large effect size was found on the two knowledge scores, t(116) = 5.68-11.58, *p* < .001, *d* = 0.57–1.36, 95% CI [0.45, 0.98].

Paired *t*-test revealed that the treatment results were stable 6 months after treatment, *t*(118) = 0.10–1.70, all *p*s < .5. One exceptions was an overall increase on RSES over time, *t*(118) = 3.56, *p* > .001.

The ANCOVAs revealed that there were no differences between the four groups 6 months after treatment on either of the treatment outcomes, *F*(1, 115) = 0.01–1.19, all *p*s > .50.

## Discussion

This trial was designed to examine potentially active treatment components in ICBT for adolescents with anxiety using a factorial experimental design. Understanding the active components of therapy can enhance our understanding of how and why treatment works and potentially enable systematic development of more effective interventions.

Overall, we found that ICBT was effective in treating adolescents with anxiety and depression, based on the two primary outcome measures BAI and BDI. Moderate to large within-group effects (intention-to-treat) were observed at post treatment, which were sustained at 6-month follow-up. The effects compare well with between-group effects reported from previous studies on ICBT for adolescents (6, 9). We found small effect sizes on the secondary outcomes of anxiety. In terms of self-esteem and quality of life, treatment effects were small, albeit close to moderate immediately after treatment and of moderate size at 6-month follow-up. The effects were somewhat smaller in the intention-to-treat analyses than in the complete case analyses. Further, we found large within-group effects with regards to knowledge gain, and this finding is in line with previous studies that have evaluated knowledge acquisition following ICBT (20–22). Similar to the previous studies, we found no association between knowledge gain and change in symptoms following treatment.

When we examined the active treatment components (intention-to-treat), we observed that learning support improved the effect of treatment on the BAI immediately after the treatment period, with a trend of improving depressive symptoms on the BDI-II. Further, learning support also enhanced outcome on the weekly measures of GAD-7, and increased knowledge gain. Thus, participants who had learning support incorporated in their ICBT treatment benefitted more from the treatment. The effects were however small or close to moderate and differences between groups were not sustained at 6-month follow up. The results thus only indicate a short-term benefit of receiving learning support. This could also be interpreted as those who received learning support had a faster rate of symptom change but that the other participants caught up over time. No effect of added chat-sessions was observed and no interaction effects between learning support and additive chat-sessions were found. Thus, when treating adolescents with anxiety in this context, ICBT with therapeutic support through email only seems to achieve effects equivalent to ICBT that includes chat-sessions.

As for the observed short-term benefits of learning support on anxiety (depression) and knowledge gain, the results could be of importance in order to improve and optimize the outcome of ICBT. Our results are in line with similar research conducted on face-to-face cognitive therapy for depressed adults (13, 24, 26). Their studies also showed that memory and learning about treatment were possible to modify by incorporating pedagogical strategies, resulting in more favorable outcomes. Although underpowered, these previous studies had effect sizes that indicated treatment benefits favoring memory support during treatment (26). Thus, patients who received strategies with intent to enhance knowledge gain tended to remember more and also showed tendencies of responding better to treatment. The effects of learning support in this study were, however, small and only significant on the BAI, and effects were not sustained at follow up. Thus, our results should be interpreted with caution.

The lack of an effect of added chat-sessions in this study was somewhat surprising, given its potential to increase adherence, acceptability and outcome in trials with adolescents (31–33). Guided ICBT has often been found to be more effective than unguided ICBT (34), but the amount of contact needed is not known, in particular for adolescents with anxiety. Our results suggest that there is a limit when additional therapeutic contact does not improve clinical outcome, and that a brief weekly dose of therapeutic support most likely is sufficient. For example, Dear et al. (70) found that guidance based on weekly supportive emails was enough for treatment effect in a transdiagnostic ICBT-treatment for adults with primary anxiety. It is possible that the chat-sessions in the present study were too demanding, i.e., might have been experienced as an extra stressor beyond working with the modules, or a distraction from the treatment content, and thus not contributing to any treatment effect during the treatment period. This might be the case especially for the participants in the treatment group who received both learning support and chat-sessions. Important to note, however, is that the findings could be sample specific and need to be evaluated further in other studies.

Further, increased knowledge levels were not associated with symptom reduction and did not predict treatment success at 6-month follow-up. The lack of association between knowledge gains and symptom reduction has been found in our previous research on adolescents with depression (22). Thus, participants can learn about explicit treatment points but in terms of symptom reduction not necessarily benefit from acquired knowledge. This could imply that gaining knowledge about CBT is in itself insufficient to improve from treatment. It is also possible that knowledge is an independent construct that is important and another way to measure outcome. Knowing is not necessarily improving or doing things differently, but is arguably crucial for improvement in CBT as psychoeducation and a clear rationale that the client understands is a prerequisite for many techniques such as exposure. Important to note, however, is that knowledge gain was measured with a multiple-choice test. Multiple-choice test requires recognition of facts, rather than active retrieval and recall of treatment content. Such memory processes might capture a more personalized form of knowledge and are thus of more practical importance. Even if the questions in the test used here were formulated as mini-vignettes, with aim to measure application and generalization of core principles and treatment content, multiple-choice tests do not necessarily measure a deeper understanding or application of treatment techniques.

The adherence rate was equal across the four treatment groups, with an average module completion rate of 64.0%. Thus, neither learning support nor added chat-sessions affected adherence in terms of modules completed. Module completion rate was somewhat lower compared to our previous ICBT studies on adolescents with depression (31, 32), but higher than what can be expected when delivering unguided internet interventions for adolescents (29, 30).

### Limitations

The findings should be interpreted in light of several limitations. First, learning support was incorporated with the intention to enhance treatment outcome through strengthened understanding and remembering the material. However, we do not know if it was this that produced the indicated effects, and more research is needed on the mechanisms of change. It is also possible that the incorporated learning strategies contributed to a stronger sense of therapeutic presence in the texts, i.e., that the modules with learning support were perceived as more flexible and responsive to the reader. Thus, we do not know if the observed effect can be referred to active learning processes, or if the incorporated learning support strategies rather give the participants a stronger sense of alliance to treatment. The importance of engaging the reader by incorporating common factors has been addressed in the context of self-help books for depression (71). The learning support condition seemed to enhance the readers’ engagement in the texts, working as a form of persuasive design (28). Incorporating persuasive design (technological strategies with the intent to engage and the expose the reader more to the content) has been found to give better outcomes. It is worth noting that the standard treatment was already fairly pedagogical in its essence, as ICBT is a highly structured treatment with modules provided on a well-designed website. The observed effects indicate that the effect of incorporating learning support in ICBT should be explored further. Using different techniques to help participants memorize treatment might be one important reason when and why ICBT works. One concern is also weather the enhanced effects are related to the fact that the modules with learning support were longer and thus exposed the participants to a higher dose of treatment content.

Another possible limitation concerning the design is the lack of control group since all groups received an active ICBT treatment. However, we already have evidence supporting that treatment is better than no treatment (e.g., (6, 9)), and it is unlikely that the observed effects are unrelated to the intervention.

Further, we used final year clinical psychology students as therapist and did not measure therapeutic adherence or competence, i.e., we did not control for whether, or with what skill, therapists delivered the theory-specified techniques of the interventions in their written feedback and support (72). Especially for the chat-sessions, this could be of potential importance, and ratings of therapeutic adherence and competence could preferably be done in future studies using chat-sessions as therapeutic support. However, the students received weekly supervision with experienced ICBT-therapists and the chat-sessions followed a semi-structured manual.

As mentioned, one limitation is how we measured knowledge in this trial and whether we really measured and covered all relevant aspects of an ICBT treatment for anxiety and depression in adolescents. Another limitation of the measures used concerns their general lack of validation in the adolescent population, with an exception of the BDI-II. This raises issues with regard to the measures’ validity to detect anxiety in a meaningful way in this population. Also, the amount of secondary outcomes were many and potentially time-consuming, which could partly explain some of the missing data at post treatment. We removed three of the secondary measures of anxiety in the 6-month follow up-assessment to lessen the burden for the respondents. We used several outcomes measures since this study was a transdiagnostic trial and we wanted to target a wide range of symptoms.

An intriguing amount of the participants were females (81%). A preponderance of females has been observed in previous trials of ICBT (31, 32) Thus, there is a need to find ways to increase the amount of males in internet trials on youths, in order to improve the generalizability of results to a young male population.

Further, we only observed short term benefits of learning support, albeit the follow-up period was short. Longer follow-ups could be conducted to investigate the long-term effects of the intervention (14).

Finally, since this study was not performed in a hospital setting we could not cross check medical records for diagnostic verification. Optimizing pharmacological treatment along with ICBT would be interesting for future studies, as to evaluate which diagnostic classes that responds best to the therapy modalities.

In spite of these limitations our findings show the importance of evaluating active components of ICBT and that learning support strategies are potentially important factors warranting further research.

## Conclusions

To our knowledge, this is one of the first studies experimentally examining active components of an ICBT treatment for adolescents with anxiety. Using a factorial design this study addresses a gap in the literature concerning why treatment works and how to optimize treatment for this population. The findings indicate that learning support strategies could play an important role in enhancing clinical outcome in the treatment of adolescent anxiety, at least in a short-term perspective. Further research is warranted to verify results and to investigate whether findings can be generalised to other diagnostic populations as well as to face-to-face therapy formats.

## Data Availability Statement

The datasets generated for this study are available on request to the corresponding author.

## Ethics Statement

Ethics approval was granted by the region of Östergötland (reg. no 2017/489-31). All participants provided informed consent online.

## Author Contributions

JB, MN, KSt, LV, EW, HÅ, KSi, GA, and MB authored the treatment material. JB, MN, KSt, LV, EW, and HÅ treated the participants. KSi, MB, and MZ were clinical supervisors during the treatment period. AR gave feedback on the treatment material and was consulted when statistical analyses were performed. AC provided medical consultation when needed. NT developed the chat-function and designed parts of the program together with GA. All authors read and provided valuable feedback on the ms.

## Funding 

This work was supported in part by a Professors grant from Linköping University (GA, PI) and in part from the Swedish Foundation for Humanities and Social Sciences, grant no. P16-0883:1 (from the Swedish Central Bank). The funding source had no involvement in the study.

## Conflict of Interest

The authors declare that the research was conducted in the absence of any commercial or financial relationships that could be construed as a potential conflict of interest.
