# Supplementary material for: The Role of Learning Support and Chat-Sessions in Guided Internet-Based Cognitive Behavioral Therapy for Adolescents With Anxiety: A Factorial Design Study
Source: Front Psychiatry. 2020 Jun 10;11:503. doi: 10.3389/fpsyt.2020.00503 (PMC7298729; doi:10.3389/fpsyt.2020.00503)
Supplement: Supplementary file 1 [file DataSheet_1.docx]

**Appendix A.** Module overview

| **Week** | **Content** | **Learning support strategies** |
| --- | --- | --- |
|  |  |  |
| **1** | Introduction to ICBT and primary anxiety and comorbid depressive symptoms, introducing the concept of ICBT and how the treatment would proceed. Psychoeducation about the symptoms and causes of anxiety from a CBT-perspective was presented. Exercises were to write history and set goals. | Rationale about the importance of active retrieval and reflecting upon one’s own learning throughout the treatment. Psychoeducation was applied on fictive cases, later followed throughout each module. The readers were asked to reflect upon how the information related to their earlier knowledge about anxiety and how to manage it. A module summary and an exercise in creating an own module summary was provided. |
| **2** | Psychoeducation about cognitive restructuring, explaining the relationship between thoughts and emotions. Exercises on how to identify and challenge negative thoughts. | Quiz about the core content in module 1, to stimulate repetition and practice remembering. Retrieval was also stimulated by asking the readers to reflect on how they would tell a significant other about the treatment content. Pictures illustrating the connection between thoughts and emotions were presented. A module summary was provided as well as an exercise in writing your own summary. |
| **3** | This module focused on behavioural activation, educating the clients about the link between behaviours and emotions. Exercises were to identify patterns of behaviour and to incorporate meaningful activities a mood-activity diary. | Quiz about the core content in module 2, praising recall when all questions were right. The readers were asked to reflect upon how core points in the module would apply on examples in their own life. Pictures illustrating the link between behaviours and emotions were provided, as well as a module summary. Exercise was to write your own summary. |
| **4** | Introduction to functional behaviour analysis and exercises on how to analyse situations that lead to undesirable and desirable consequences. | Quiz about the core content in module 3, praising recall when all questions were right. Pictures and a video illustrating functional behaviour analysis were provided, as well as a module summary. Exercise was to write your own summary. |
| **5** | This module focused on psychoeducation and exercises about exposure, i.e. how to gradually challenge one’s fears by approaching situations that have been previously avoided. | Quiz about the core content in module 4, praising recall when all questions were right. The readers were encouraged to continuously reflect upon the information provided and if they recognize themselves while reading it. Pictures and a short video illustrating exposure were provided, as well as a module summary. Exercise was to write your own summary. |
| **6** | This module continued to focus on graded exposure and how to cope with difficulties when trying to apply it. Exercise was to keep doing the exposure. | Quiz about the core content in module 5, raising recall when all questions were right. The readers were asked to reflect upon the information and how they would advise a significant other to act in the same situation. A module summary was provided and the exercise to write an own module summary. |
| **7** | Psychoeducation about affect regulation and self-esteem, focusing on how to cope with negative emotions and how to increase experiences of self-worth. Exercise in accepting and allowing negative emotions to occur without acting upon them. | Quiz about the core content in module 6, praising recall when all questions were right. Asked to reflect upon what was new to them in the information content and how they would summarize it to a significant other. A short video about self-worth, pictures and a module summary was provided. Exercise in writing an own module summary. |
| **8** | The final module included treatment summary and psychoeducation about relapse prevention. Exercise in writing about what you’ve learned and describing the treatment in your own words. | Quiz about the core content in module 7, praising recall when all questions were right. Summary of the whole treatment provided. |
|  |  |  |
|  |  |  |
