# Supplementary material for: The Role of Learning Support and Chat-Sessions in Guided Internet-Based Cognitive Behavioral Therapy for Adolescents With Anxiety: A Factorial Design Study
Source: Front Psychiatry. 2020 Jun 10;11:503. doi: 10.3389/fpsyt.2020.00503 (PMC7298729; doi:10.3389/fpsyt.2020.00503)
Supplement: Supplementary file 2 [file DataSheet_2.docx]

**Below you will find 16 questions regarding what you know about Cognitive Behaviour Therapy (CBT).**

This test is about what you currently know about CBT. Also, please rate how certain you are about your answer, that is, if you are guessing, feeling pretty certain, or are quite certain.

**Note**! Choose the option that you think is the *most* correct, according to CBT. Different answers can of course be the right one depending on person and situation. Here, however, we would like you to respond to what you think is **most important according to CBT.**

**1. According to CBT , what happens if you avoid things that evokes painful emotions?**

1… The painful emotions will gradually decrease over time.

2… The painful emotions can momentarily become worse.

3… *The painful emotions will linger in the long run.*

How confident are you in your answer?

- I’m guessing, I’m pretty certain, I’m quite certain.

**2. According to CBT , what happens if you escape from a harmless situation that raises anxiety?**

1… It is a quick and helpful way to reduce anxiety.

2… The anxiety increases momentarily, but it is a good strategy in the long run.

3… *It is a quick way to reduce anxiety, but the anxiety will probably return the next time you are in similar situations.*

How confident are you in your answer?

- I’m guessing, I’m pretty certain, I’m quite certain.

**3. According to CBT , what is the most helpful way to handle negative thoughts?**

1… Try to think positively as often as you can

2… *Try to allow negative thoughts but then try to challenge them*

3… Try to ignore negative thoughts and distract yourself with something else

**4. Can anxiety be dangerous?**

1… Yes, it is dangerous since it can lead to heart diseases or strokes

2… No, it is not dangerous, as long as it does not become too strong

*3... No, it is not dangerous, but can feel very unpleasant*

**5.When trying to manage depressive feelings and tiredness, which strategy is most effective, in the long run?**

1 ... Rest properly and try to sleep as much as you can

2 ... Try to think more positively, avoiding getting stuck in negative thoughts

3 ... *Try to do what you usually like doing, even though you do not feel like doing it in the moment.*

**6. Emma suffers from low self-esteem, which prevents her from joining a theater class. What advice would CBT give her?**

1 ... She needs to try to cheer herself up. If she can assure herself that she is good at theater, she can join a class without feeling bad about herself

2 ... She needs to try to accept that low self-esteem is a part of her personality and difficult to change. She could try to do other things than joining a theater class.

3. *She needs to try to challenge her low self-esteem and try to join a theater class anyway*

How confident are you in your answer?

- I’m guessing, I’m pretty certain, I’m quite certain.

**7. Molly is afraid of going downtown when the city is crowded with people. According to CBT, what could she try to do?**

1... Call someone who talks with her and calms her down while she runs her errands

2... Try to hurry up while thinking of something else, so that the situation passes quickly

3... *Try to gradually challenge the fear and go down town anyway*

How confident are you in your answer?

- I’m guessing, I’m pretty certain, I’m quite certain.

**8. Marcus is just about to attend an important class, but becomes nervous, gets palpitations and chills. According to CBT, what could he try?**

1 ... Skip class and go home, so that his anxiety does not get worse

2 ... Try to think of something else so that his body calms down

3 ... *Try to challenge the anxiety and enter class even if it feels very uncomfortable*

How confident are you in your answer?

- I’m guessing, I’m pretty certain, I’m quite certain.

**9. Alice finds it scary to eat lunch with her best friends at school, so she ends up eating lunch by herself. What could she try doing, according to CBT?**

1 ... Give it time and wait until it feels good to eat lunch with her friends

2 ... Be nice to herself and just keep avoiding eating lunch with her friends if it feels uncomfortable

3 ... *Try to challenge the discomfort and start eating lunch with them anyway*

How confident are you in your answer?

- I’m guessing, I’m pretty certain, I’m quite certain.

**10. Peter is tired even though he sleeps a lot. He does not have the energy to do things he used to. What would be most helpful for him to try, according to CBT?**

1 ... Make sure to rest properly, so that he gradually becomes more energized

2 ... *Gradually start doing things he used to enjoy doing, even though he feels tired*

3 ... Gradually try to think more positively so that he feels a little happier

How confident are you in your answer?

- I’m guessing, I’m pretty certain, I’m quite certain.

**11. Edvin is afraid of embarrassing himself in front of his friends and rarely joins them when they are hanging out, even though he wants to. What could he try to do, according to CBT?**

1 ... Try to suppress the fear and calm himself down by assuring himself that they are his best friends

2 ... *Try to act against the fear and hang out with them anyway*

3 ... Try to do other things where he does not feel anxious about himself

How confident are you in your answer?

- I’m guessing, I’m pretty certain, I’m quite certain.

**12. Johan walks into a store and experiences a sudden feeling of discomfort and notice that his heart is beating rapidly. This often happens when he is around other people. According to CBT, what strategy could be helpful to try?**

1… Try to get out of store as fast as possible, so his body can calm down.

2… Try to ignore how the body feels and hurry through the shopping

3… *Try to act against the anxiety and continue to shop as planned*

**13. Sofia is at a party and feels socially excluded. She is thinking that nobody likes her and that her friends find her boring. What is most helpful for her to do, according to CBT?**

1 ... Try to think more positively, for example that she is a good friend and that the party is quite funny

2 ... Try to go home so she does not have to feel socially excluded and boring

3 ... *Try to let the thoughts be and try to shift focus on what is actually going on around her*

**14. Anna is about to hold a lecture to her class, but she feels very nervous and plans to pretend to have a cold in order to avoid it. How could one perceive this situation, according to CBT?**

1 ... It is okay if she avoids holding the lecture since the situation makes her so anxious.

2 ... It is okay to lie since Anna suffers from anxiety and may need some peace and calm.

3 ... *If she does not try to hold the lecture it could prevent her from noticing that the situation may not be as terrible as she fears it to be.*

How confident are you in your answer?

- I’m guessing, I’m pretty certain, I’m quite certain.

**15. Michael often feels depressed and dull. What is the best thing he can do in order to feel better, according to CBT?**

1 ... Try to do what he feels like in the moment

2 ... Try to stay at home, sleep and wait for the depressive feelings to pass

3 ... *Try to do things that he used to feel good about doing, even if it does not feel good to do it in the moment.*

How confident are you in your answer?

- I’m guessing, I’m pretty certain, I’m quite certain.

**16. Sara worries about her future, she often thinks about what she should do and gets stuck while searching the web for various options on education and travels. What would CBT recommend her to do?**

1 ... Try to think that everything will be fine

2 ... Try to talk about her worry with many people, until she feels calm

3 ... *Try to pause and shift focus to what is happening inside her, in the moment, instead of searching the web.*

How confident are you in your answer?

- I’m guessing, I’m pretty certain, I’m quite certain.
